# Pilot study of neurologic toxicity in mice after proton minibeam therapy

**DOI:** 10.1038/s41598-020-68015-0

**Published:** 2020-07-09

**Authors:** John G. Eley, Awalpreet S. Chadha, Caio Quini, Elisabeth G. Vichaya, Cancan Zhang, James Davis, Narayan Sahoo, Jaylyn Waddell, Dominic Leiser, F. Avraham Dilmanian, Sunil Krishnan

**Affiliations:** 1 Department of Radiation Oncology, Vanderbilt University School of Medicine, 2220 Pierce Avenue, Preston Research Building B1003, Nashville, TN 37232 USA; 2 Department of Radiation Oncology, The University of Texas MD Anderson Cancer Center, 1515 Holcombe Boulevard, Houston, TX 77030 USA; 3 Department of Symptom Research, The University of Texas MD Anderson Cancer Center, 1515 Holcombe Boulevard, Houston, TX 77030 USA; 4 Department of Radiation Oncology, University of Maryland School of Medicine, 655 West Baltimore Street, Baltimore, MD 21201 USA; 5 Department of Pathology, Stony Brook Medicine, 101 Nicolls Road, Stony Brook, NY 11794 USA; 6 Department of Radiation Physics, The University of Texas MD Anderson Cancer Center, 1515 Holcombe Boulevard, Houston, TX 77030 USA; 7 Department of Pediatrics, University of Maryland School of Medicine, 655 West Baltimore Street, Baltimore, MD 21201 USA; 8 Departments of Radiology, Neurology, and Radiation Oncology, Stony Brook Medicine, 101 Nicolls Road, Stony Brook, NY 11794 USA; 9 Department of Radiation Oncology, Mayo Clinic Florida, 4500 San Pablo Road S., Jacksonville, FL 32224 USA

**Keywords:** Biophysics, Cancer, Oncology

## Abstract

Proton minibeams (MBs) comprised of parallel planar beamlets were evaluated for their ability to spare healthy brain compared to proton broad beams (BBs). Juvenile mice were given partial brain irradiation of 10 or 30 Gy integral dose using 100 MeV protons configured either as BBs or arrays of 0.3-mm planar MBs spaced 1.0 mm apart on center. Neurologic toxicity was evaluated during an 8-month surveillance: no overt constitutional or neurologic dysfunction was noted for any study animals. Less acute epilation was observed in MB than BB mice. Persistent chronic inflammation was noted along the entire BB path in BB mice whereas inflammation was confined to just within the MB peak regions in MB mice. The potential neurologic sparing, possibly via reduced volume of chronic inflammation, offers a compelling rationale for clinical advancement of this proton technique.

## Introduction

The mechanisms of radiation-induced brain injury are pervasive and include^1–3^: vascular-endothelial damage leading to cerebral ischemia and breakdown of the blood–brain barrier, cell death in oligodendrocytes and oligodendrocyte precursors leading to insufficient myelin, activation of astrocytes that cause gliosis and breakdown of the blood–brain barrier, activation of microglia, chronic oxidative stress, depletion of neural stem cells in hippocampus, and direct neural damage. Many of the above mechanisms together lead to severe quality of life issues for brain cancer survivors including: loss of vision or hearing, anxiety, depression, or chronic fatigue, cognitive impairment, low intelligence quotient (IQ) score, academic impairment, and impaired executive functioning.

Our research aims to mitigate these neurologic side effects by combining two distinct fields of radiation research: light-ion therapy and minibeam therapy. While particle therapy (e.g., using protons or carbon ions) in many cases offers dosimetric advantages compared with megavolt photon therapy, still, it does not generally spare healthy tissue in the beam entrance channel. While that problem can be mitigated by using multiple beam entrance angles, that solution leads to greater volumes of healthy tissue in the irradiated field. Our method undertakes a new conceptual direction, which builds on the early experience of synchrotron X-ray minibeams.

In that field of research, Zeman et al.^4^ initially demonstrated that the mouse cerebellum tolerated irradiation up to 10,000 Gy deuterons by single 25-micron beams without showing any histological lesion or cavitation. Later, Slatkin et al.^5^ showed that rat cerebellum tolerated parallel, thin planes (37 microns) of synchrotron X-ray radiation up to 250 Gy in-beam dose. The excitement produced by these findings led to the initiation of a larger number of studies with X-ray microbeams (< 300 microns) and minibeams (300–700 microns) at both the NSLS and the European Synchrotron Research Facility (ESRF, Grenoble, France). Recently, the method’s tissue sparing has been confirmed also using carbon-nanotube X-rays turned into minibeams^6^, by 20 MeV microchannel protons in the mouse ear^7^, and by 100 MeV proton minibeams in the rat brain^8^. A recent study by Dilmanian et al.^9^ experimentally demonstrated that the concepts of minibeam therapy and proton therapy could be combined in a manner to exploit both therapeutic strategies at once: A proton field can be delivered in a segmented array of unidirectional minibeams, which is expected to spare shallow tissues, but will itself scatter into a tumoricidal, broad-beam, due to multiple Coulomb scattering, before reaching a therapeutic target.

The purpose of this study was to test the hypothesis that proton minibeam therapy would show a difference in biologic damage to normal mouse brain compared to (conventional) proton broad-beam therapy while achieving identical dose at deeper depths where a tumor would hypothetically lie. To test our hypothesis, we irradiated mice using 100 MeV protons traversing laterally through the brain, configured as either broad beams or minibeam arrays. Animals were followed for 8 months and subjected to cognitive studies. At the study termination, brain tissue was analyzed to characterize the chronic neurologic changes produced.

## Methods

### Experimental proton conditions and physical measurements

**Figure 1:**
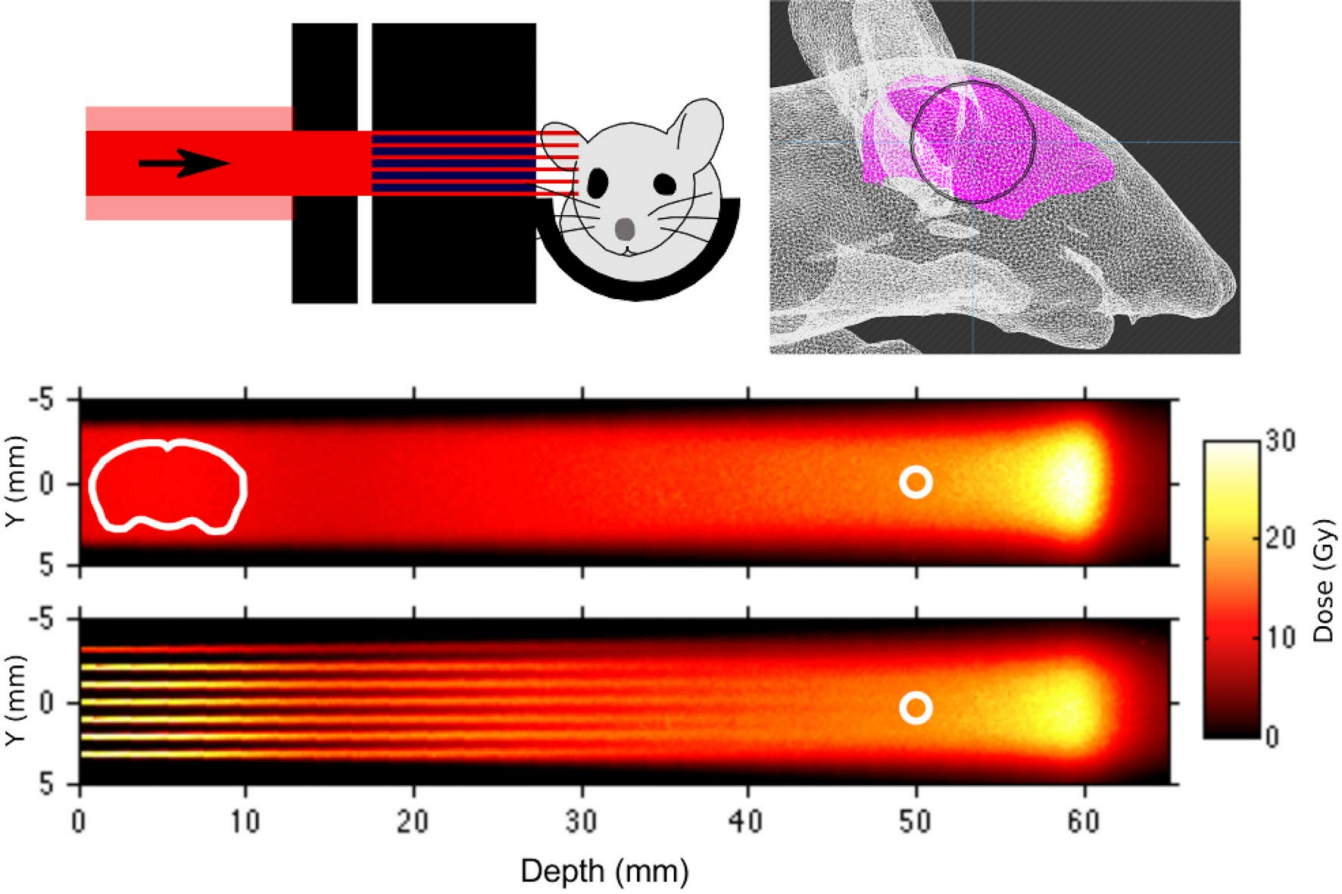
Diagram (top left) of experimental setup indicating relative position of the proton beam, the broad-beam collimator, the multislit collimator, and the animal. Beam’s eye view (top right) of irradiation field boundary (black circle), defined by the circular broad-beam collimator, centered approximately on the midbrain/hippocampus (brain indicated in pink) for one mouse that was imaged using micro computed tomography. Radiochromic film measurements (bottom) of broad-beam (BB10) and minibeam (MB10) experimental conditions with approximate position of mouse brain indicated by the white contour; the small white circles at 50-mm depth indicate the dose normalization points, where BB and MB conditions provided the same mean dose.

Irradiations were carried out using the experimental beamline at the UT MD Anderson Proton Therapy Center (Houston, TX). Monoenergetic, 100-MeV proton pencil beams were used for all irradiations. The experimental setup is shown in Fig. 1. For both broad-beam and minibeam irradiations, a 2-cm-thick brass collimator having a 7-mm-diameter circular aperture was used to produce a solid radiation beam with circular cross section. For minibeams only, an additional multislit tungsten collimator (5-cm thick in the beam direction) was used to divide the beam into an array of planar minibeams with 0.3-mm width and 1.0-mm spacing (on center). Prior to animal irradiations, physical dosimetry was established using Gafchromic EBT3 radiochromic film (Ashland, Covington, KY), cross calibrated to ion chamber measurements using the PTW Advanced Markus Chamber (PTW, Freiburg, Germany) following the International Atomic Energy Agency TRS-398 dosimetry protocol (Vienna, 2000). For the broad-beam and minibeam irradiations; both methods were normalized to provide identical dose at a depth of 5 cm, where the minibeam array has fully merged into a broad beam (see Fig. 1). Peak-to-valley ratios were defined as the ratio of minibeam-peak dose at a certain depth to the local valley dose at the same depth, i.e., the valley refers to the local minimum dose between 2 adjacent minibeams in the array.

### Mouse irradiations

Beams were designed to shoot through the mouse brain laterally. As shown in Fig. 1, beams were centered approximately on the hippocampus by its relation to relevant surface landmarks (namely the eye and top of the scalp) determined from a computed tomogram (CT) of one mouse. Thus, nearly 2/3 of the brain was irradiated, including the cortex and hippocampus, key regions for brain development and cognition. Juvenile, male mice (C57BL/6J, *n* = 24, post-natal day 28) were randomly assigned to 5 experimental arms: no radiation (Sham, *n* = 6), broad-beam 10 Gy (BB10, *n* = 6), minibeam 10 Gy (MB10, *n* = 6), broad-beam 30 Gy (BB30, *n* = 3), and minibeam 30 Gy (MB30, *n* = 3). Animal experiments were reviewed and approved by the UT MD Anderson Cancer Center Institutional Animal Care and Use Committee (Protocol #1163).

### Cognitive studies

The Novel Object Task^10,11^ was performed at 1, 2, and 3 months after irradiation to assess perirhinal-cortex dependent declarative memory^10–12^. Testing was carried out in a quiet room using a white empty testing arena of 20 × 20 cm^2^ floor size. We used 5 min exploration time for the two identical initial objects, a 5 min retention interval, and 5 min exploration time of the novel object, which was also placed in a novel location. Videos were recorded and interaction times were scored manually with a stopwatch by an investigator blind to the study conditions. The discrimination index (DI) was calculated using the interaction times (*t*) as

$$ {\text{DI}} = {{{1}00\% t\left( {{\text{novel}}} \right)} \mathord{\left/ {\vphantom {{{1}00\% t\left( {{\text{novel}}} \right)} {\left[ {t\left( {{\text{novel}}} \right) + t\left( {{\text{initial}}} \right)} \right]}}} \right. \kern-\nulldelimiterspace} {\left[ {t\left( {{\text{novel}}} \right) + t\left( {{\text{initial}}} \right)} \right]}}. $$

DI greater than 50% was interpreted to mean the mouse correctly remembered the initial object and was therefore more interested to explore the novel object in the novel place. In general, a lower score was interpreted to indicate impaired recognition memory.

The Social Recognition Task^13,14^ was performed at 1, 2, and 3 months post-irradiation. Testing was carried out in the animal’s home cage. Each mouse was exposed to an initial juvenile conspecific for 5 min, allowed to rest for 1 h retention interval, and afterwards exposed to the initial conspecific and a novel conspecific for 5 min. The discrimination index was calculated and interpreted as described above.

The Morris Water Maze Task^15^ was carried out at 3 months post-irradiation. A 1.5**-**m-diameter tank was used, filled with water with temperature of 22 ± 1 °C. Quadrants A, B, C, and D were demarcated using solid geometric figures (cues) (triangle, square, horizontal bars, and vertical bars) attached to the interior tank walls above the waterline. A transparent acrylic escape platform was submerged in the center of Quadrant D. Over the course of 2 days, 15 training trials were carried out to teach the mice to swim the tank and to find the escape platform and to learn its location. Escape latency was timed with a stopwatch. On day 3 of testing, an alternate-starting-position trial was carried out to test spatial learning. Additionally, a 2-min probe trial was carried out with the hidden platform removed. Probe swim trials were analyzed using a software tool developed in MATLAB (Version R2018b, MathWorks, Natick, MA) that analyzed swim paths and scored the fraction of time spent searching in the target quadrant.

### Histologic investigations

At 8 months after irradiation, brain tissues were harvested and fixed by cardiac injection-perfusion with 4% paraformaldehyde. 5-micron sagittal slide sections were taken 5-mm off midline (towards the beam entrance) for immunohistochemistry staining: haematoxylin and eosin (H&E), glial fibrillary acidic protein (GFAP), nerve/glial-antigen 2 (NG2), nestin, and cluster of differentiation 31 (CD31). Slides were scored by a blinded, board-certified neuropathologist using four categories: No immunoreactivity, Mild immunoreactivity, Moderate immunoreactivity, or Strong immunoreactivity. In addition, digital brightfield microscope images (Axio Imager M2, Carl Zeiss Microscopy GmbH, Jena, Germany) were acquired for each section for a hippocampal ROI as well as for the entire whole-brain sections. Image analysis was performed using Fiji (ImageJ, v1.51 u, 64 bit Windows) software. For quantification, images were processed in analogy to Andy’s Algorithms (Law, 2017): first a ROI inside the brain tissue and in the radiation beam path was selected. A Fiji color deconvolution with H&E DAB filter was applied. Next a Gaussian blur was used to reduce noise and the images were converted to an 8-bit image. A threshold was set over all ROIs using the ImageJ “default” threshold function. The images were then converted to a binary image and the percentage of stained area was determined. In addition, we performed sensitivity analysis of our histologic findings by varying the ROI size and by varying the scoring metric (fractional area versus mean signal intensity). These sensitivity analyses are included in Supplement 1.

### Statistical analysis

For analysis of serial behavioral testing data, we used Repeated Measures ANOVA with Bonferroni Correction for Multiple Comparisons. For analysis of other endpoints, without repeated measures, we utilized the two-sample t-test to compare study arm MB10 against BB10 and to compare study arm MB30 against BB30. All tests were two-sided with α = 0.05. *p*-values less than 0.05 were considered significant. All values are reported as means ± 95% confidence intervals.

### Ethical approval

All methods were carried out in accordance with relevant guidelines and regulations.

## Results

The experimental irradiation conditions and corresponding film measurements are shown in Fig. 1. Film measurements indicated peak entrance doses to be 10.0 ± 0.1 Gy, 33.1 ± 0.5 Gy, 30.0 ± 0.3 Gy, and 99.3 ± 1.5 Gy for arms BB10, MB10, BB30, and MB30, respectively. For minibeam irradiations, the peak-to-valley ratio at the (skin) entrance was 72.0 ± 1.1. For both broad beams and minibeams, doses were equal at the normalization point at 5-cm depth in plastic: 14.2 Gy for BB10 and MB10 and 42.6 Gy for BB30 and MB30. A detailed analysis of the dose statistics at variable depths is included in Table 1 for BB and MB exposure conditions. Remarkably, over the 8-month study period, no animals in any arm showed signs of radiation illness or motor impairment, and all animals gained weight normally, despite the high single-fraction doses.

**Table 1:** Dose statistics at variable depths for BB10 and MB10 experimental conditions, corresponding to radiochromic film measurements shown in Fig. 1.

Depth (cm)	BB10 dose (Gy)	MB10 peak dose (Gy)	PVR
0.0	10.0 ± 0.1	33.1 ± 0.5	72.0 ± 1.1
0.5	10.9 ± 0.3	30.9 ± 1.3	67.3 ± 2.9
1.0	9.7 ± 0.3	21.9 ± 0.7	47.7 ± 1.5
1.5	9.0 ± 0.2	16.8 ± 0.4	36.6 ± 1.0
2.0	9.4 ± 0.3	15.7 ± 0.6	6.1 ± 0.7
3.0	12.1 ± 0.2	14.3 ± 0.4	1.7 ± 0.1
4.0	14.2 ± 0.7	13.8 ± 0.6	1.1 ± 0.2
5.0	17.8 ± 0.4	16.0 ± 0.7	1.0 ± 0.0
BP	36.2 ± 1.0	27.4 ± 0.8	1.0 ± 0.0

Mean and 95% confidence intervals given for each depth, averaged from five manual point-dose measurements at each depth. Peak-to-valley ratios (PVR) are the ratio of the peak dose and the valley dose (minimum dose between adjacent minibeams) at each depth. For reference, the mouse brain is approximately 1-cm in lateral width, thus the depths from 0 to 1 cm are representative of the animal exposure conditions. The Bragg Peak (BP) depth was approximately 6 cm. The dosimetric conditions for BB30 and MB30 can be derived by multiplying these dose values by a factor of 3.

### Cognitive studies

**Figure 2:**
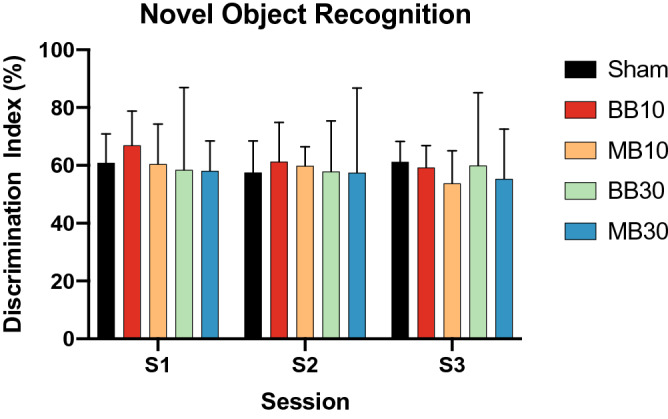
Discrimination Index scores for the Novel Object Task for each experimental group, for sessions S1, S2, and S3 at 1, 2, and 3 months after irradiation, respectively. Bars and error bars show means and 95% confidence intervals. No indications of cognitive impairment were observed.

**Figure 3:**
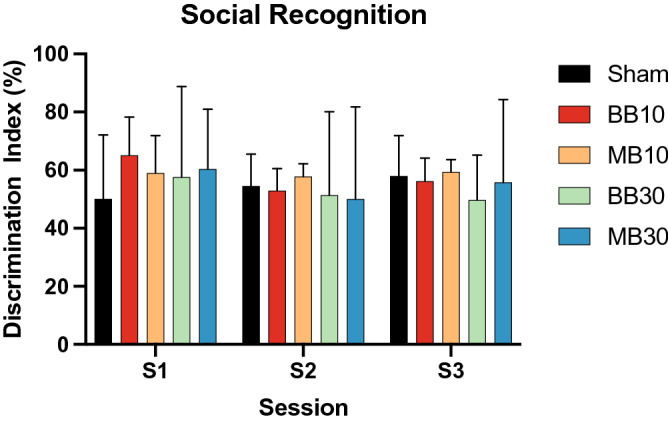
Discrimination Index scores for the Social Recognition Task for each experimental group, for sessions S1, S2, and S3 at 1, 2, and 3 months after irradiation, respectively. Bars and error bars show means and 95% confidence intervals. At later timepoints S2 and S3, higher-dose groups BB30 and MB30 showed slightly lower mean scores than other groups (not significant).

**Figure 4:**
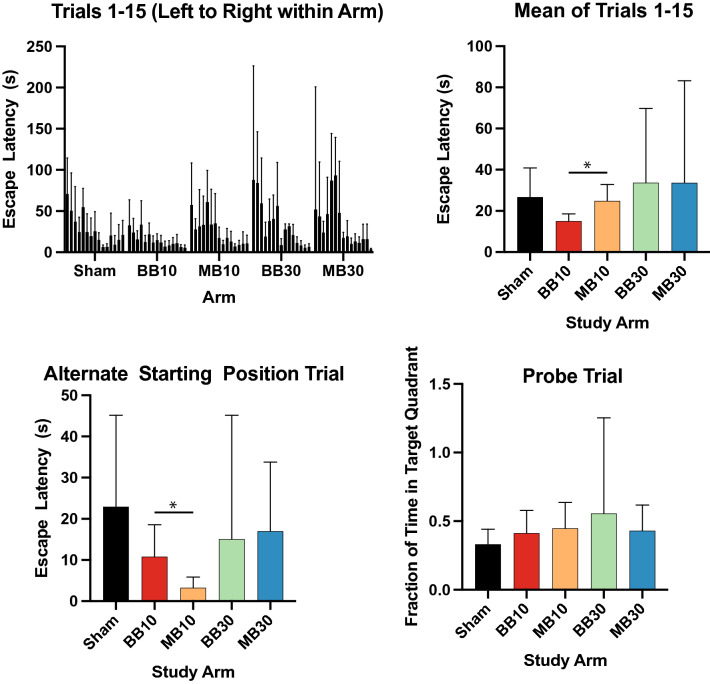
Results from the Morris Water Maze Task. For each study arm, (top left) escape latencies are seen to decrease over the 15 training trials. Mean escape latencies over all 15 trials are shown at top right, lower for BB10 than MB10 (*p* = 0.0246). Escape latency for the Alternate Starting Position Trial (bottom left), which required spatial navigation, is seen to be lower for MB10 than for BB10 (*p* = 0.0395). Probe trial results (bottom right) show the fraction of time each mouse spent swimming in the Target Quadrant, where the hidden platform was normally located; no significant differences were noted in the Probe Trial. Bars and error bars show means and 95% confidence intervals.

Results from the Novel Object Task are shown in Fig. 2. No significant differences were seen between MB10 versus BB10 or between MB30 versus BB30. Results from the Social Recognition Task are shown in Fig. 3. No significant differences were seen in DI scores between MB10 and BB10 or between MB30 and BB30. Figure 4 shows results from the Morris Water Maze Task. The mean escape latency, averaged over the 15 training trials, was significantly lower for study arm BB10 compared with MB10 (*p* = 0.0246). Mean escape latencies over the 15 trials were 26.7 ± 10.9, 15.1 ± 2.7, 24.9 ± 6.0, 33.7 ± 16.5, and 33.7 ± 22.6 s for arms Sham, BB10, MB10, BB30, and MB30, respectively. For the alternate-starting-position trial, which aimed to explicitly test hippocampal-dependent spatial learning based on the visual cues, the escape latency was significantly lower for arm MB10 compared with BB10 (*p* = 0.0395). Mean escape latencies for the alternate-starting-position trial were 22.9 ± 16.9, 10.8 ± 5.9, 3.3 ± 2.0, 15.1 ± 13.7, and 17.0 ± 7.7 s for arms Sham, BB10, MB10, BB30, and MB30, respectively. For the probe trial, no significant differences were seen between any study arms compared.

### Histologic investigations

**Figure 5:**
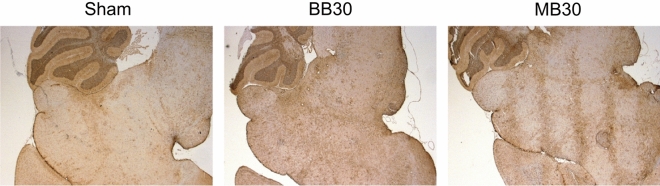
GFAP stains for Sham, BB30, and MB30. Patterned, moderate gliosis corresponding to the minibeam dose pattern is seen for MB30 versus a diffuse region of moderate gliosis seen for BB30. Comparably mild gliosis is seen for Sham and in the gaps between minibeams in MB30.

**Figure 6:**
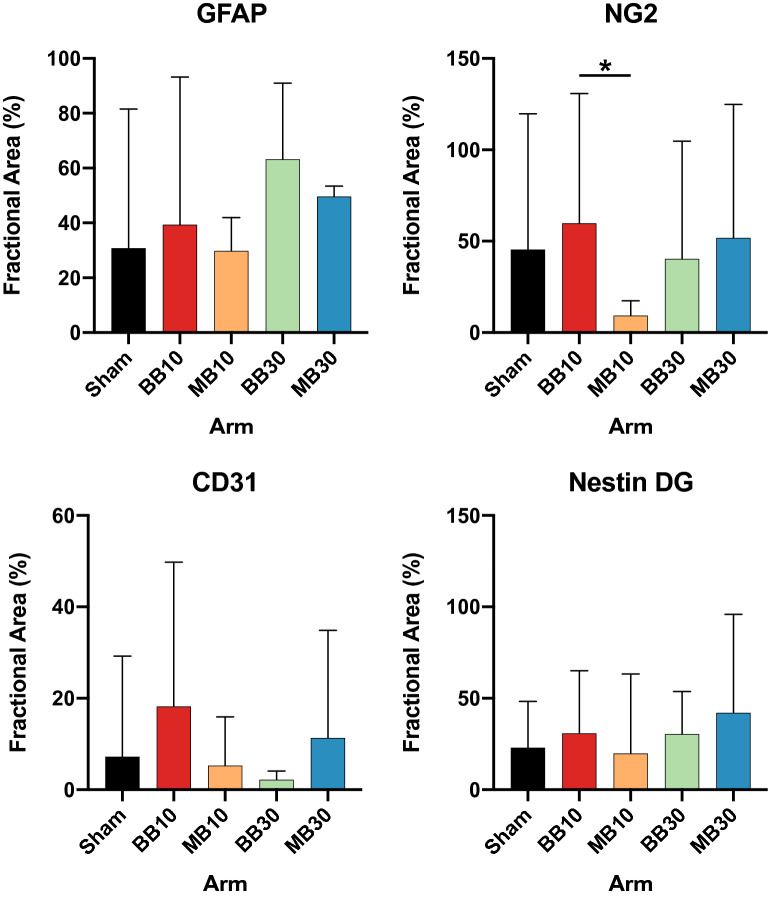
Fraction of stained area in regions of interest (ROI) for GFAP, NG2, CD31, and Nestin. The ROI for GFAP analysis was placed in the center of the irradiated brain region and showed a higher mean value for BB10 than MB10 (not significant) and higher mean value for BB30 than MB30 (not significant), which we interpret to indicate a larger area of activated astrocytes (gliosis) for BB versus MB mice, as seen in Fig. 5. The ROI for NG2 analysis was identical to that of GFAP. Analysis for NG2 showed a lower mean value for MB10 than for BB10 (*p* = 0.0385); however, no significant differences were observed between MB30 and BB30. Differences between study arms after CD31 and Nestin labeling were not significant. Bars and error bars show means and 95% confidence intervals.

GFAP stains revealed systematic variations in gliosis among study arms. Our neuropathologist observed Moderate immunoreactivity/gliosis in arms BB10, BB30, and MB30, while Mild immunoreactivity/gliosis was seen in arms Sham and MB10. The fractional area of brain showing GFAP-labeling was less on average in the MB conditions, compared to the BB conditions (cf. Figs. 5 and 6). While these average differences in GFAP-labeling across the irradiated region ROIs were not significant, MB animals consistently showed spatial confinement of gliosis to the minibeam pattern (cf. Fig. 5 and Supplement 1), with regions of tissue sparing between minibeams, whereas BB animals had a diffuse pattern of gliosis without tissue sparing. This implies the volume of neuroinflammation induced by radiation may be reduced with proton minibeams compared to proton broad beams. Analysis for NG2 showed a lower mean value for MB10 than for BB10 (*p* = 0.0385); however, no significant differences were observed between MB30 and BB30. CD31 and Nestin labeling in the dentate gyrus (DG) did not reveal significant differences in vascularity/neurogenic status among study arms. Microscope images for each sample are included in Supplement 1. Sensitivity testing (Supplement 1) revealed that GFAP, NG2, and Nestin DG were minimally sensitive to ROI choice or metric (fractional area versus mean intensity), while CD31 and Nestin CA1 findings were more sensitive to ROI choice and metric.

### Skin damage

**Figure 7:**
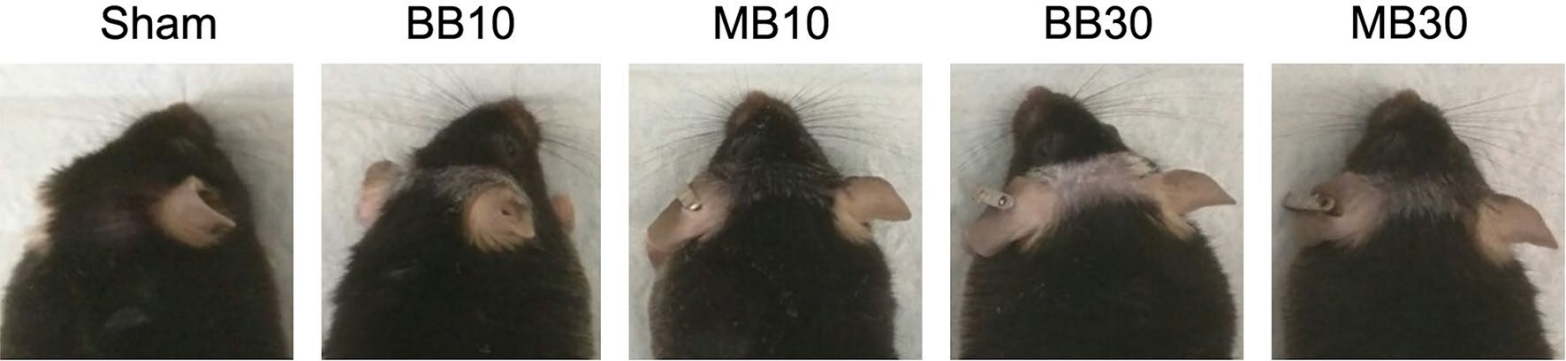
Photographs of epilation at 3 months post irradiation for different study arms. For every animal, epilation was higher for mice exposed to broad beams (BB10 and BB30) compared to those exposed to minibeams (MB10 and MB30, respectively).

An ad hoc categorical scale was defined to score epilation into 4 categories: None (Level 0), Mild (Level 1), Moderate (Level 2), and Severe (Level 3), corresponding to visible levels of epilation of < 5%, 5–30%, 30–70%, and > 70%, respectively. Despite the coarseness of the scale, the epilation trends were unanimous within study arms. Severe (Level 3) epilation was seen in arm BB30. Moderate (Level 2) epilation was seen in arms BB10 and MB30. Mild (Level 1) epilation was seen in arm MB10, and no (Level 0) epilation was seen in arm Sham. Figure 7 shows photographs of epilation for a representative animal for each study group at 3 months after irradiation.

## Discussion

While the behavioral studies, taken as a whole, were not conclusive regarding the difference in cognitive side effects after proton minibeam therapy compared to proton broad-beam therapy, the pathologic findings in brain revealed confinement of reactive gliosis to the minibeam spatial pattern. With regard to toxicity, we found that all animals survived all study arms without apparent motor deficits and gained weight normally, including the group MB30, which had peak entrance doses of 99.3 ± 1.5 Gy in single fraction exposures traversing the cortex and hippocampus. Although skin damage was not the primary focus of this study, we found clear evidence of advantage for proton minibeams over proton broad beams. This finding is in agreement with the recent results from Girst et al.^7^. In our study, the reduced skin damage also implies that a physical collimator might be acceptable for clinical use, even if magnetically focused minibeams are theoretically preferable.

For the behavioral tests with the water maze, the neural substrates used to successfully locate the hidden platform depend on the training procedure used. Initial training was conducted using a consistent starting point. Performance was faster in mice irradiated with BB. However, when the start location was varied, it was revealed that MB mice were faster to locate the escape platform. We speculate one hypothesis that might explain these findings is that disruption of the hippocampus by radiation exposure could lead to a striatal-based learning strategy^16–18^ that might be effective and faster than other groups to learn the location of an escape platform in relation to a fixed starting position, whereas an intact hippocampus would acquire encoding of spatial cue information that would lead to faster escape when the starting position was varied. However, due to the small number of animals studied in this pilot, we cannot rule out the possibility that these behavioral findings will prove to be irreproducible.

One strength of this study was that we demonstrated that a peak-to-valley ratio of 72:1 could be attained using protons at therapeutic energies relevant for human brain tumors using a metallic, multislit collimator. This approach can thus likely be easily implemented at other proton therapy facilities. Another advantage of this work was that our method avoids the tremendous technical hurdle of the interleaved carbon minibeam method of Dilmanian et al.^19^, which requires interleaving of the minibeam treatment fields with a precision of 100 microns or better and allows no body motion between subsequent beams. Compared with previous minibeam studies, in this study, testing was performed with doses of radiation that are similar to those used in clinical radiosurgery. In addition, we used an immune-intact, general mouse model and followed animals for 8 months, allowing observation for late effects and full realization of the phenotypic response.

Our study had several limitations. First, due to its pilot nature, the number of animals was kept small. This was intended by design, as no previous publications could be used to estimate expected sample means and variances. Second, the study only investigated CNS damage in the shallow part of the proton field, where proton minibeams remained spatially distinct. Thus, no evidence other than physical (film) measurements is presented to imply equal tumor control of the methods. Third, we did not investigate the method in comparison with photon therapy. We intentionally chose broad-beam proton exposures as our control rather than photon exposures to limit the number of free variables in the study and to avoid potential confounds.

Looking forward, the next steps will likely be to confirm the findings in a higher number of animals and in multiple species. As shown computationally by Dilmanian et al.^9^, other ions such as ^4^He, ^7^Li, and ^12^C might also be valuable to extend the depths of sparing beyond that of protons, due to less elastic scattering. Also, our method is theoretically compatible with intensity modulated particle therapy, since the beams can be modulated upstream of a multislit collimator, though simulations and experiments are needed to establish that next step.

In conclusion, our findings indicate that proton minibeam therapy can offer reduced volumes of neuroinflammation in mouse brain compared to proton broad-beam therapy. The spectrum of behavioral tests showed no conclusive differences in cognitive function despite a factor of roughly 3.3 increase of the in-beam dose using minibeams. These findings, together with our secondary finding of reduced skin damage using proton minibeam therapy instead of proton broad-beam therapy, inspire that continued investigation of this novel therapy is warranted.

## Supplementary information

Supplementary file1 (PDF 33219 kb)

## Data Availability

The datasets generated during and/or analyzed during the current study are available from the corresponding author on reasonable request.
