# Supplementary material for: Pilot study of neurologic toxicity in mice after proton minibeam therapy
Source: Sci Rep. 2020 Jul 9;10:11368. doi: 10.1038/s41598-020-68015-0 (PMC7347840; doi:10.1038/s41598-020-68015-0)
Supplement: Supplementary file 1 — Supplementary file1 (PDF 33219 kb) [file 41598_2020_68015_MOESM1_ESM.pdf]

Supplement 1

Pilot Study of Neurologic Toxicity in Mice after Proton Minibeam Therapy

John G Eley PhD[1], Awalpreet S Chadha MD[2], Caio Quini PhD[2], Elisabeth G Vichaya PhD[3], Cancan Zhang MD[4], James Davis MD[5], Narayan Sahoo PhD[6], Jaylyn Waddell PhD[7], Dominic Leiser MD[4], F Avraham Dilmanian PhD[8], and Sunil Krishnan MD[2]*

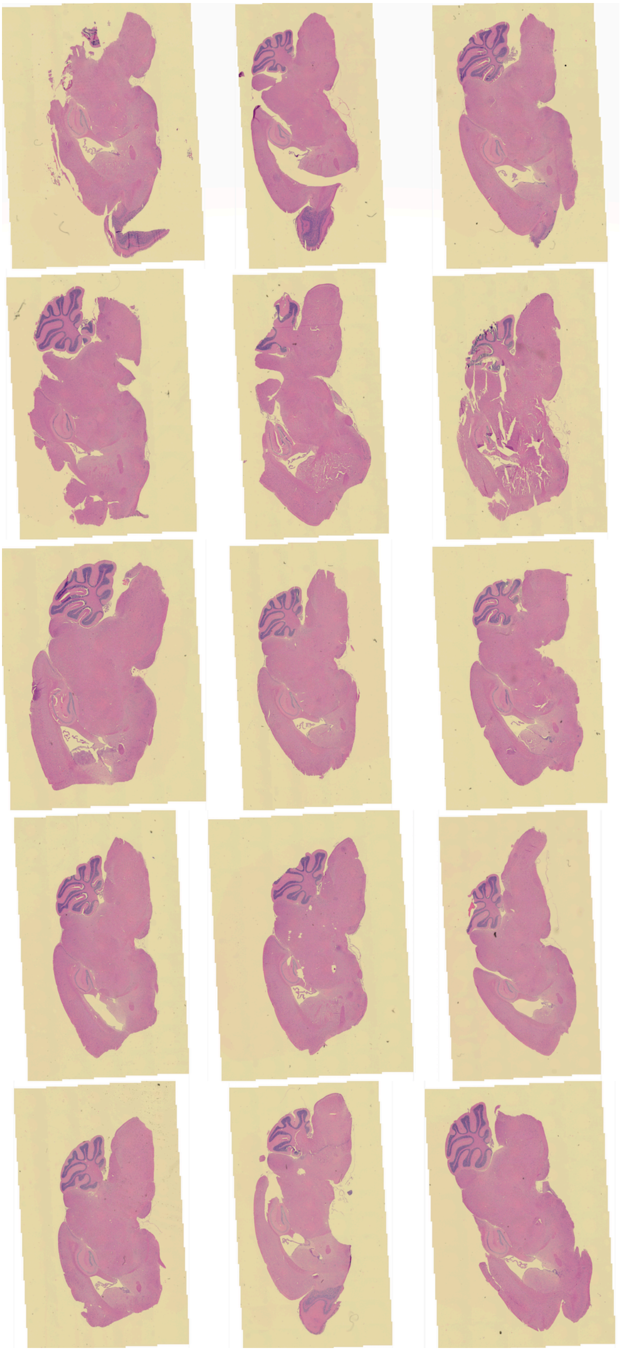

Figure S1-1. Hematoxylin and eosin (H&E) staining did not reveal pathologic differences among study arms. 3 animals are grouped (left-to-right) for each study arm, from top to bottom in the order of: Sham, BB10, MB10, BB30, and MB30.

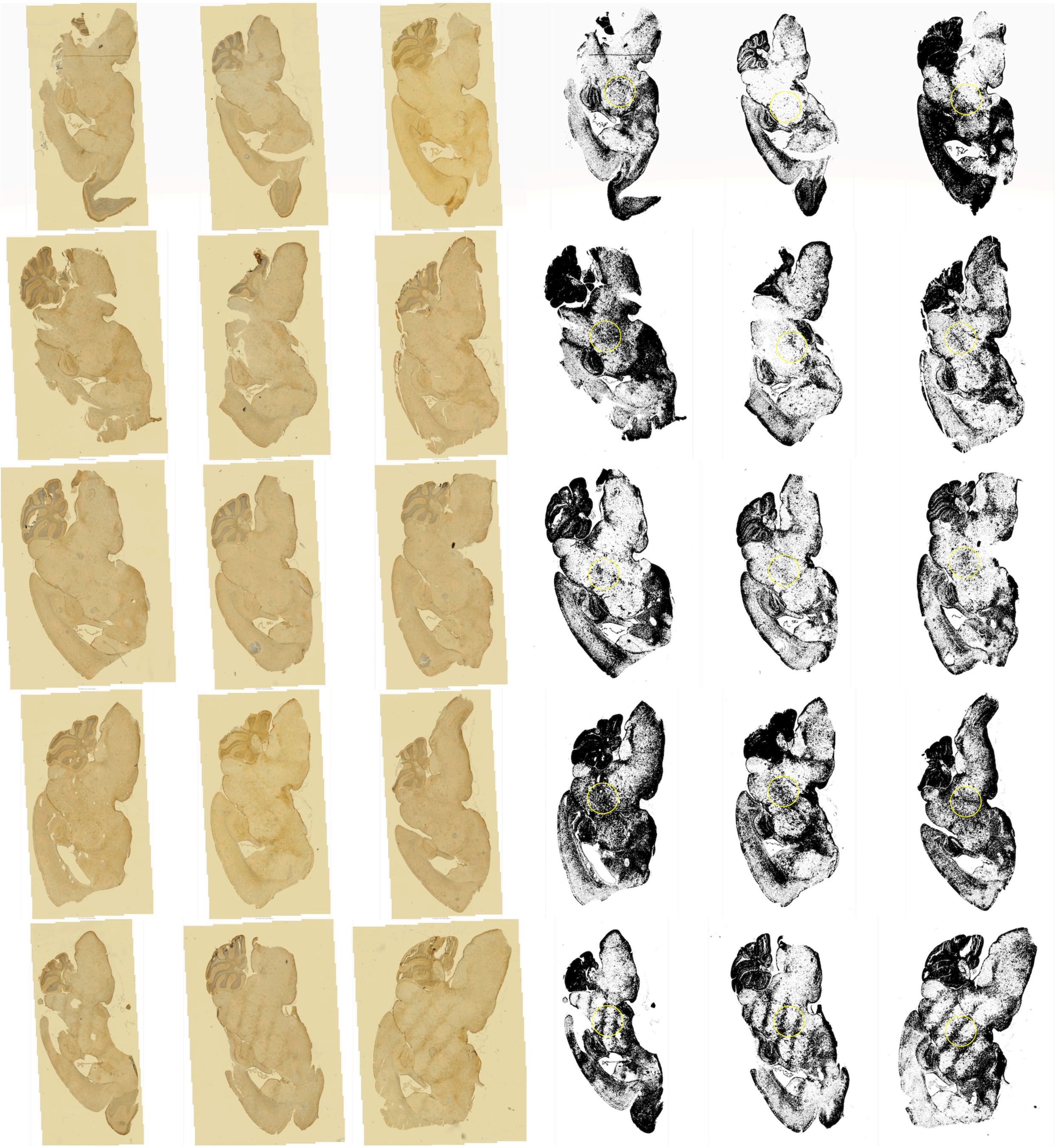

Figure S1-2. GFAP staining (left panel) revealed pathologic differences among study arms, discussed in the main manuscript. 3 animals are grouped (left-to-right) for each study arm, from top to bottom in the order of: Sham, BB10, MB10, BB30, and MB30. The counted area is shown (right panel) as a binary mask, where black regions are counted as regions of GFAP labeling and interpreted as volumes of activated astrocytes or gliosis. The ROI is shown for each animal as a small yellow circle.

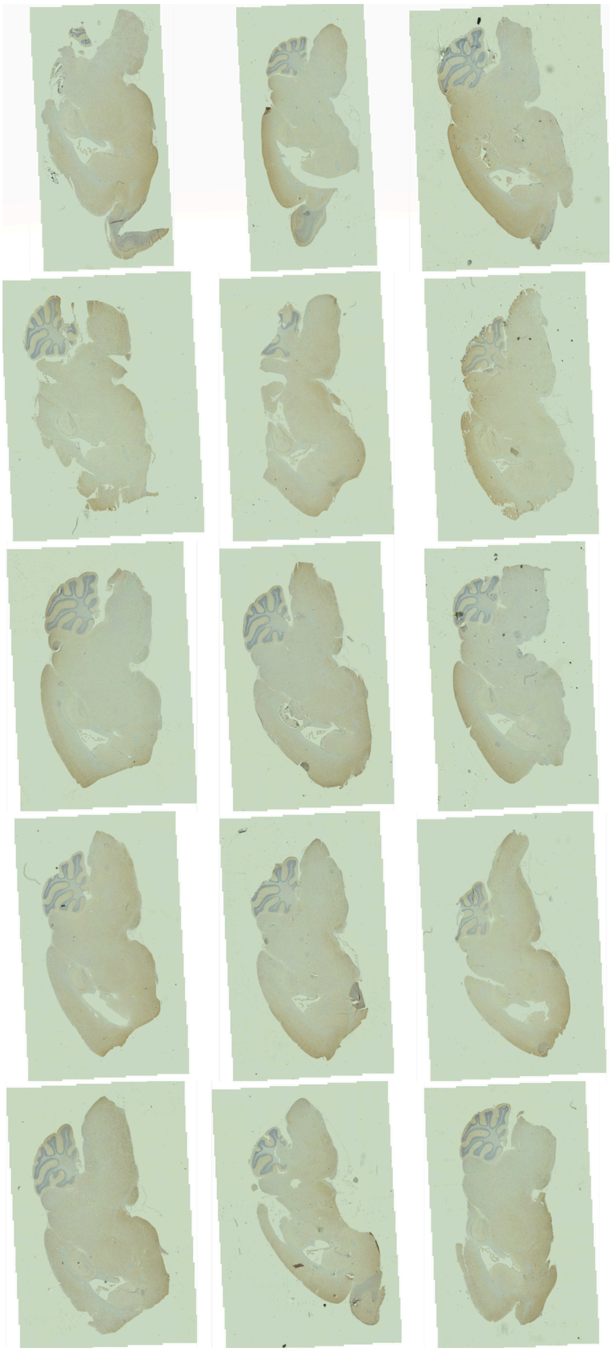

Figure S1-3. NG2 staining did not reveal differences in oligodendrocyte precursor cell density among study arms. 3 animals are grouped (left-to-right) for each study arm, from top to bottom in the order of: Sham, BB10, MB10, BB30, and MB30.

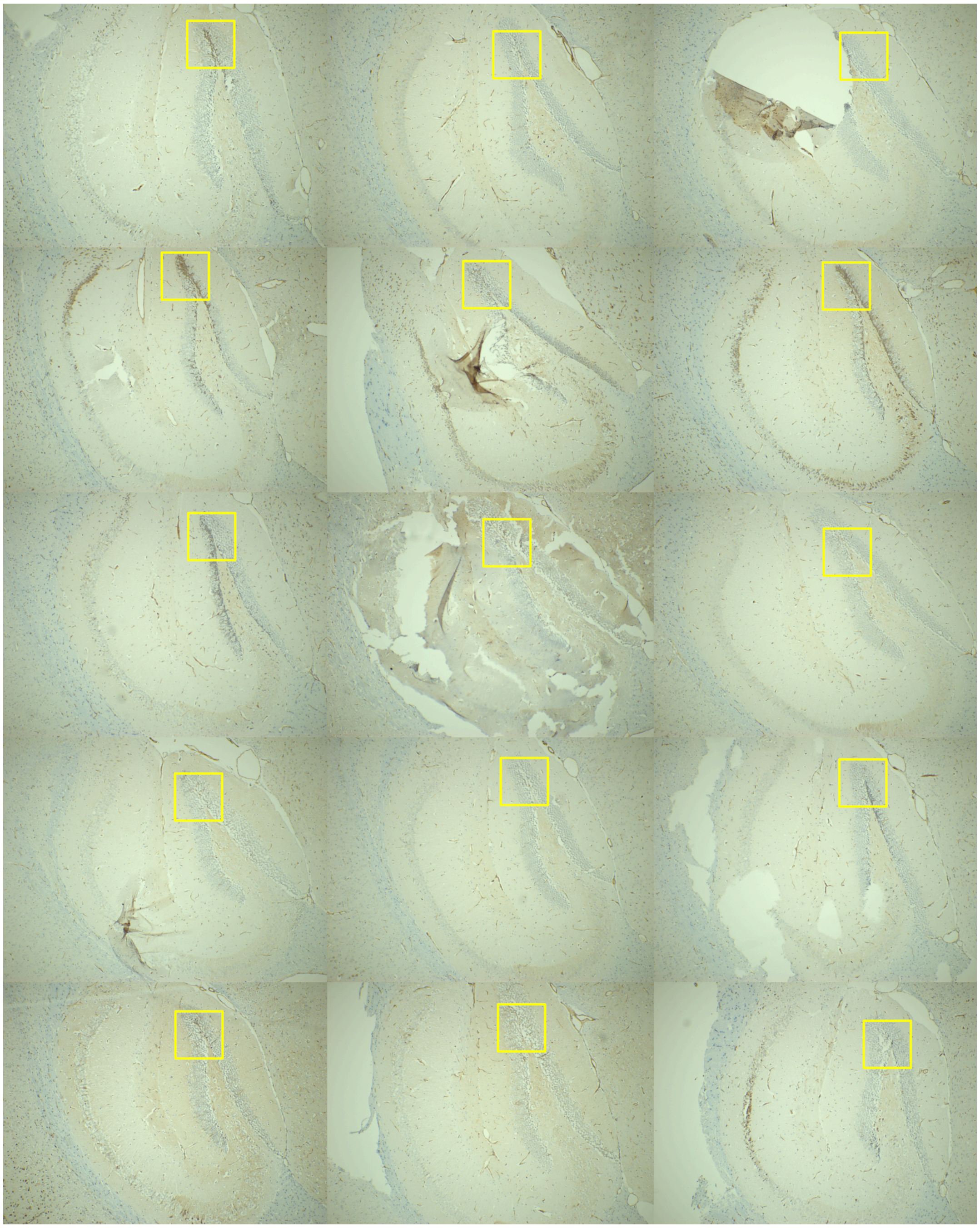

Figure S1-4. CD31 staining did not reveal differences in endothelial cell density in the DG among study arms, as discussed in the manuscript. 3 animals are grouped (left-to-right) for each study arm, from top to bottom in the order of: Sham, BB10, MB10, BB30, and MB30.

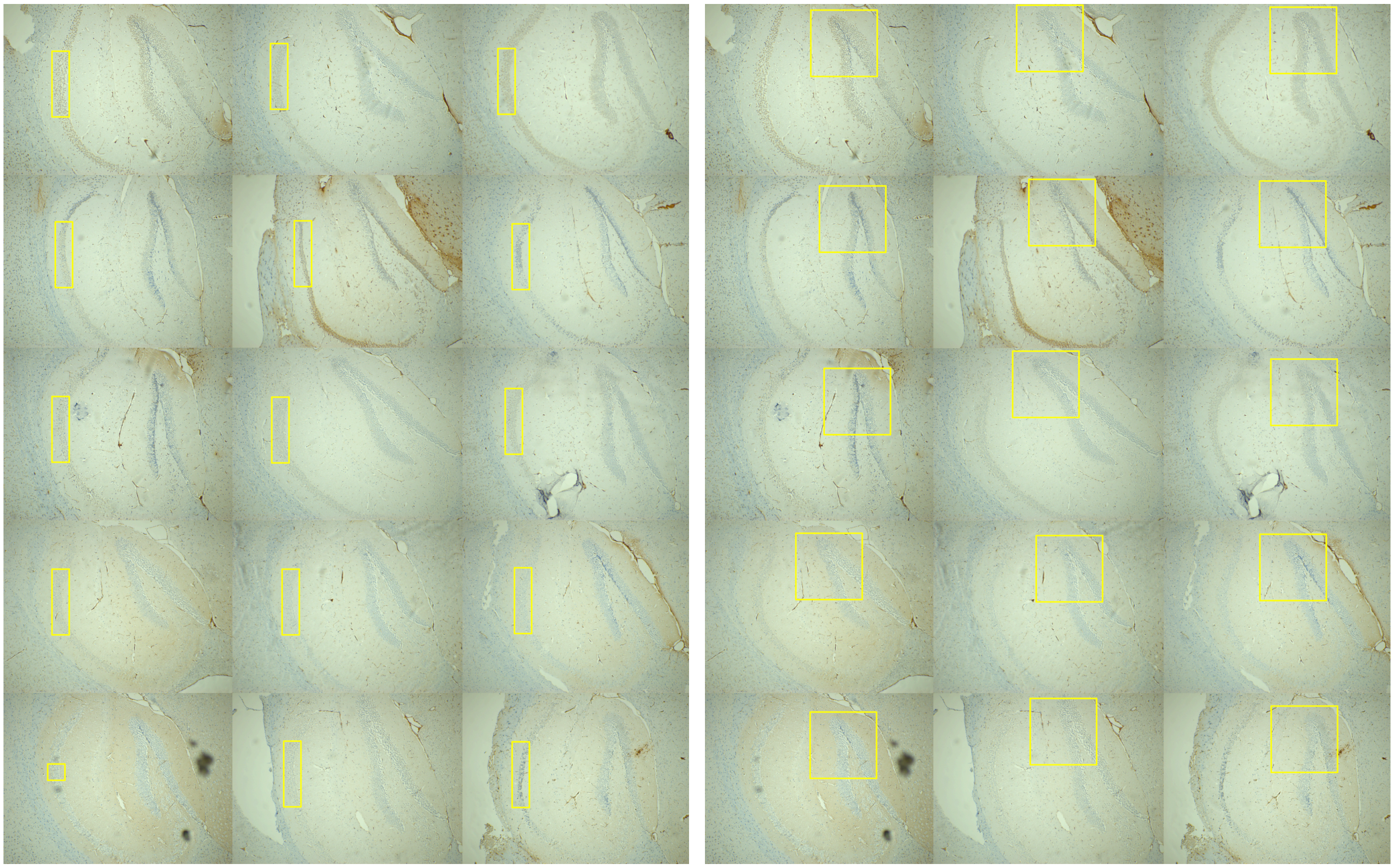

Figure S1-5. Nestin ROIs in CA1 (left panel) revealed differences among study arms, discussed in the main manuscript. Nestin ROIs in DG (right panel) did not show differences among study arms. 3 animals are grouped (left-to-right) for each study arm, from top to bottom in the order of: Sham, BB10, MB10, BB30, and MB30.

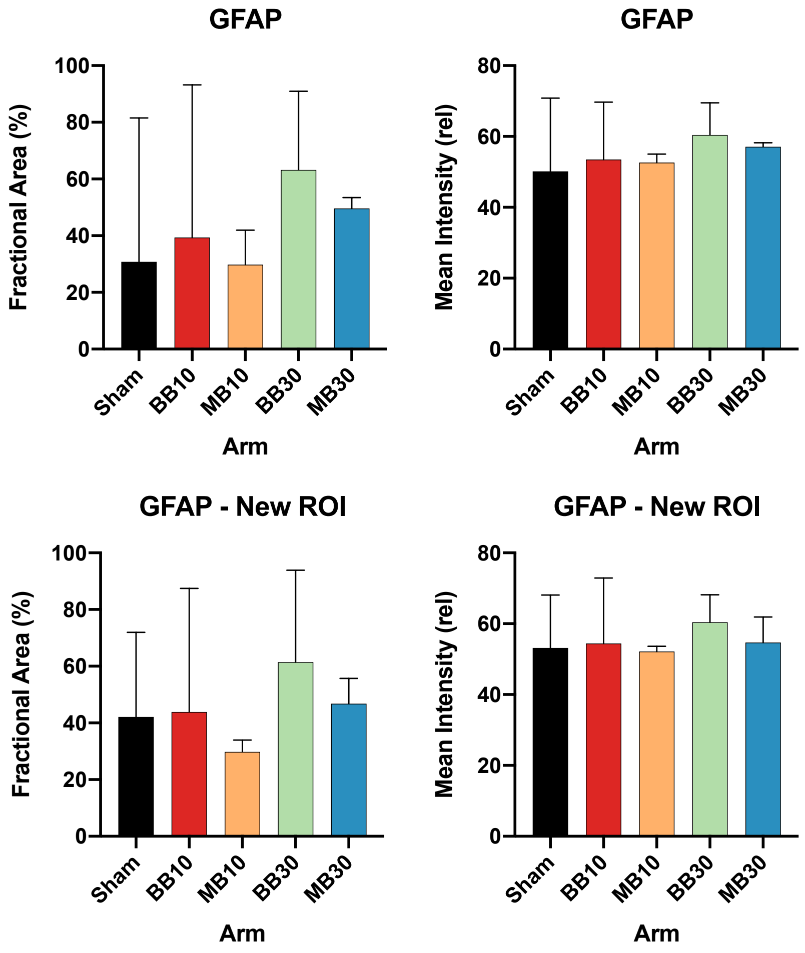

Figure S1-6. Sensitivity of GFAP analysis to region of interest (ROI) choice and metric for analysis, i.e., fractional area versus mean intensity. Fractional area scored in the original ROI, as shown in Figure S1-2 (top left). Mean intensity scored in original ROI (top right). Fractional area scored in New ROI, a circular ROI with 2 times the diameter of the original ROI, centered in the same location as the original ROI (bottom left). Mean intensity scored in the New ROI (bottom right). MB groups exhibit lower mean values of GFAP labeling than BB groups, for each dose level, irrespective of metric or ROI selection. Despite the trend, no significant differences were found.

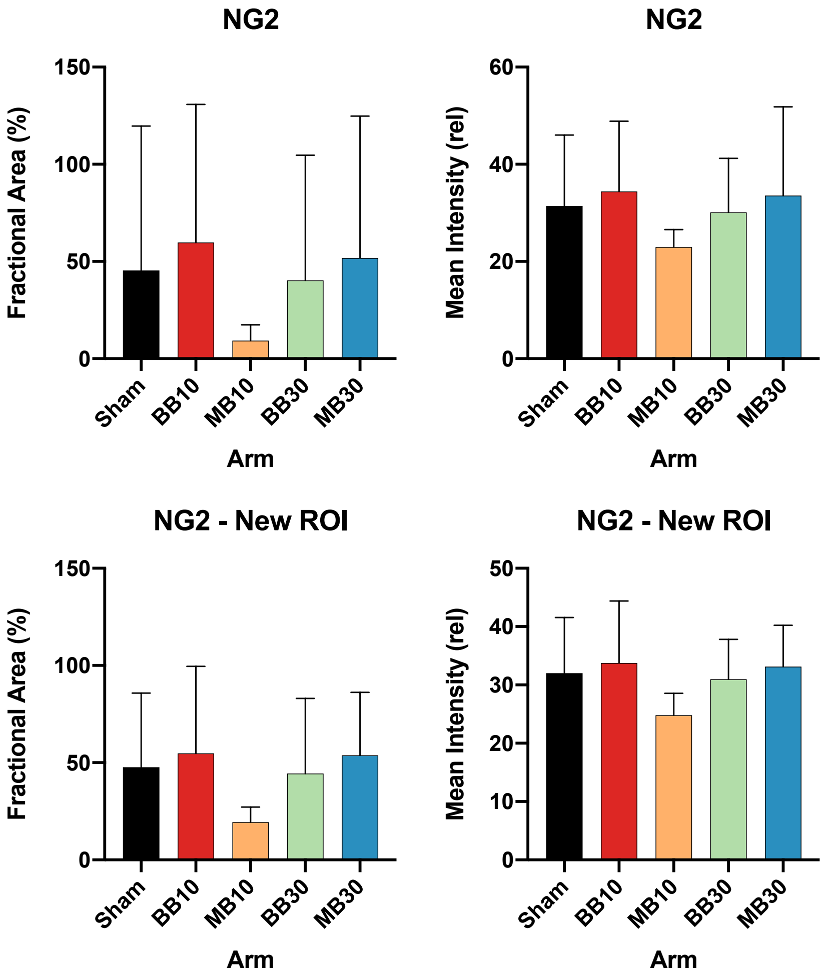

Figure S1-7. Sensitivity of NG2 analysis to region of interest (ROI) choice and metric for analysis, i.e., fractional area versus mean intensity. Fractional area scored in the original ROI, as shown for NG2 in Figure S1-2 (top left). Mean intensity scored in original ROI (top right). Fractional area scored in New ROI, a circular ROI with 2 times the diameter of the original ROI, centered in the same location as the original ROI (bottom left). Mean intensity scored in the New ROI (bottom right). At 10 Gy, MB showed significantly lower mean values of NG2 labeling than BB, irrespective of metric or ROI selection. At 30 Gy, differences between MB and BB were insignificant and similar to Sham values, again with minimal sensitivity to metric or ROI selection.

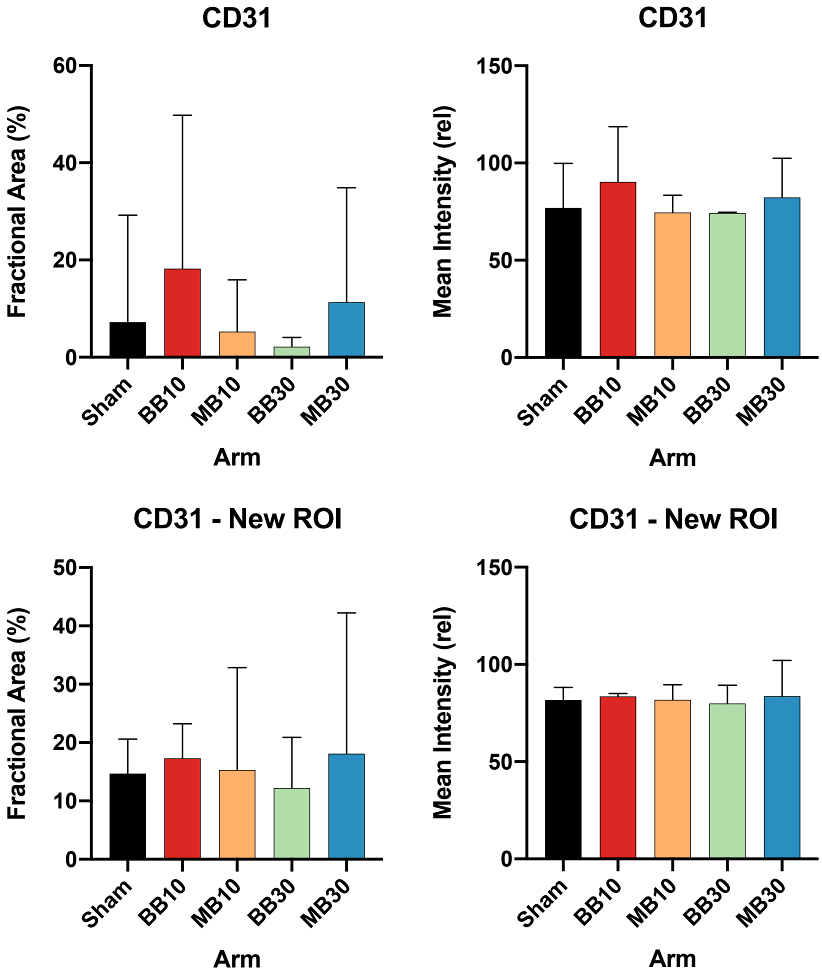

Figure S1-8. Sensitivity of CD31 analysis to region of interest (ROI) choice and metric for analysis, i.e., fractional area versus mean intensity. Fractional area scored in the original ROI, containing the Dentate Gyrus as shown in Figure S1-4 (top left). Mean intensity scored in original ROI (top right). Fractional area scored in New ROI, containing the entire image of the hippocampus seen in Figure S1-4 (bottom left). Mean intensity scored in the New ROI (bottom right). At 10 Gy, MB shows lower mean values of CD31 labeling than BB, irrespective of metric or ROI selection. At 30 Gy, the trend is reversed, with BB showing lower mean values of CD31 labeling. Overall, no significant differences were observed.

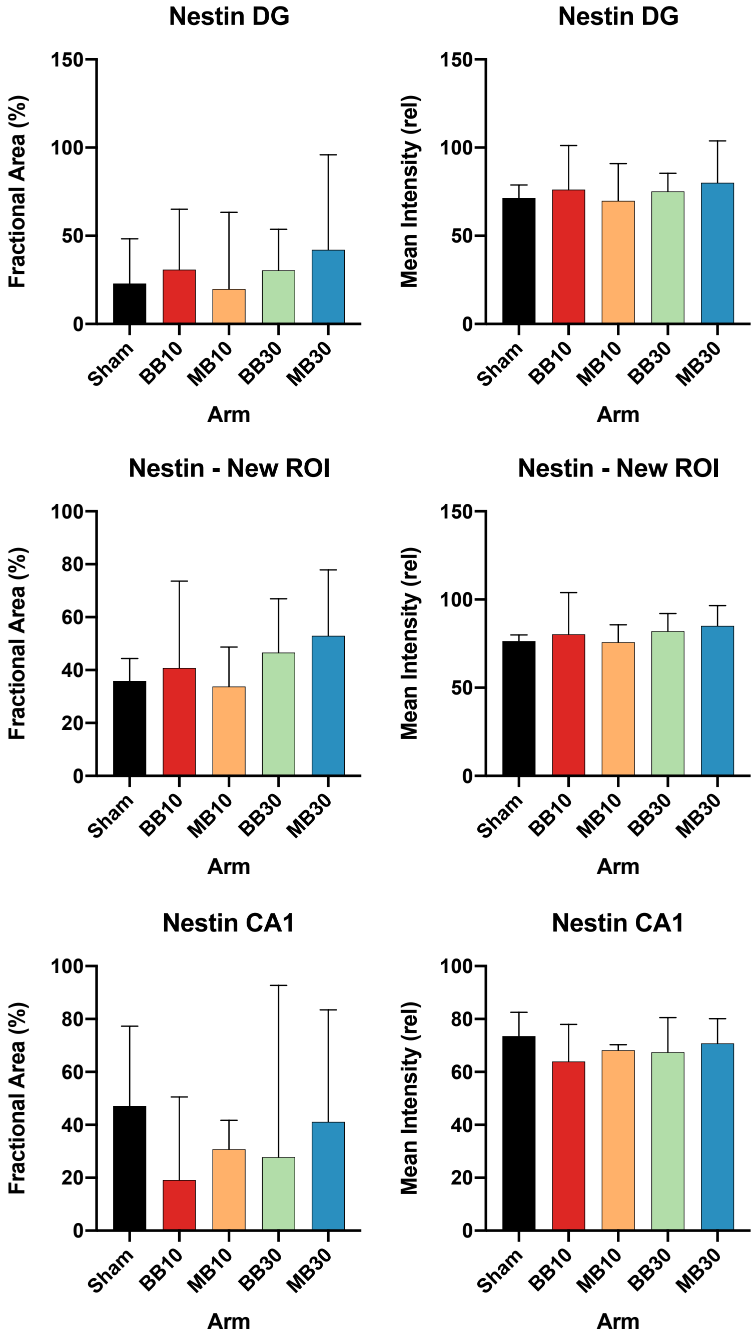

Figure S1-9. Sensitivity of Nestin analysis to region of interest (ROI) choice and metric for analysis, i.e., fractional area versus mean intensity. Fractional area scored in the original DG ROI, containing the Dentate Gyrus (DG) as shown in Figure S1-5 (top left). Mean intensity scored in original DG ROI (top right). Fractional area scored in New ROI, containing the entire image of the hippocampus seen in Figure S1-5 (middle left). Mean intensity scored in the New ROI (middle right). Fractional area scored in the original CA1 ROI, as shown in Figure S1-5 (bottom left). Mean intensity scored in original CA1 ROI (bottom right). The New ROI for CA1 is

the same as the New ROI for the DG, thus the data in the middle row are also used for sensitivity testing for CA1. Findings in the DG were minimally sensitive to ROI or metric. Findings in CA1 were sensitive to ROI choice.
